# Perception of current life situation and coping strategies in patients at the Narcological Clinic in Azerbaijan

**DOI:** 10.1192/j.eurpsy.2022.2128

**Published:** 2022-09-01

**Authors:** E. Bityutskaya, N. Baghirova-Karimli, N. Lebedeva

**Affiliations:** 1 Lomonosov Moscow State University, Faculty Of Psychology, Moscow, Russian Federation; 2 Psychological Scientific Research Institute, Department Of Corporate Cooperation And Projects, Baku, Azerbaijan; 3 Moscow Metropolitan Governance University, Diagnostics Department, Moscow, Russian Federation

**Keywords:** perceived life situation, coping strategies, goal

## Abstract

**Introduction:**

Studies show that coping skills are factors in successful rehabilitation.

**Objectives:**

The research is aimed to study the drug users` perception of the current life situation and their coping strategies.

**Methods:**

Patients at the Free Narcological Clinic in Azerbaijan (n=46; 37 men, 9 women, aged 18 to 59) participated in our study after 10-12 days (stage 1) of a rehabilitation program. We used a structured interview, projective drawings (“The image of change”, “My difficult life situation”), and a questionnaire, “Appraisal Criteria of the Difficulty of a Life Situation”. The control group consisted of 35 non-drug users. Content analysis and t-test were used.

**Results:**

While the majority of patients (77%) failed to describe any coping strategy they use in difficult life situations, all of them assessed their difficult life situation as under control—an outlook that was significantly more optimistic than the evaluation of the control group (р=0.009). The most frequently mentioned life goals were avoiding the problem (39%) and regaining health (37%). However, analysis of obstacles to achieving life goals shows that 49% of drug users mentioned no obstacles or indicated that “there are no obstacles”. Drug addiction as an obstacle is mentioned in only 4% of the responses; “environment of drug addicts” occurs as the main obstacle in 20%.

**Conclusions:**

Drug users in stage 1 of the rehabilitation program have an unrealistic sense of control, few coping strategies, and do not perceive drug addiction as posing а serious obstacle to achieving their life goals. Funding: The study was funded by RFBR, project number 20-013-00838.

**Disclosure:**

No significant relationships.

